# GCGACNN: A Graph Neural Network and Random Forest for Predicting Microbe–Drug Associations

**DOI:** 10.3390/biom14080946

**Published:** 2024-08-05

**Authors:** Shujuan Su, Meiling Liu, Jiyun Zhou, Jingfeng Zhang

**Affiliations:** 1 College of Computer and Control Engineering, Northeast Forestry University, Harbin 150040, China; sushujuan@nefu.edu.cn; 2 Lieber Institute, Johns Hopkins University, Baltimore, MD 21218, USA; jiyun.zhou@libd.org; 3 School of Computer Science, The University of Auckland, Auckland 1142, New Zealand; jingfeng.zhang9660@gmail.com

**Keywords:** graph convolutional network, graph attention network, two-dimensional convolutional neural network, microbiome–drug associations

## Abstract

The interaction between microbes and drugs encompasses the sourcing of pharmaceutical compounds, microbial drug degradation, the development of *drug resistance genes*, and the impact of *microbial communities* on host drug metabolism and immune modulation. These interactions significantly impact drug efficacy and the evolution of drug resistance. In this study, we propose a novel predictive model, termed GCGACNN. We first collected microbe, disease, and drug association data from multiple databases and the relevant literature to construct three association matrices and generate similarity feature matrices using Gaussian similarity functions. These association and similarity feature matrices were then input into a multi-layer Graph Neural Network for feature extraction, followed by a two-dimensional Convolutional Neural Network for feature fusion, ultimately establishing an effective predictive framework. Experimental results demonstrate that GCGACNN outperforms existing methods in predictive performance.

## 1. Introduction

Microbes exist either as single-celled organisms or as colonies of cells and are composed of *bacteria*, *archaea*, *fungi*, *viruses*, and *protozoa* [1]. The constituents of the microbiota—*bacteria*, *viruses*, and *eukaryotes*—have been shown to interact with one another and with the host immune system in ways that influence the development of disease [2]. A variety of microbes exist throughout the human body and have a fundamental role in human health [3]. For example, the microbiota is an essential component of immunity and a functional entity that influences metabolism and modulates drug interactions. Furthermore, ecological dysbiosis or imbalance of microbes may also lead to other diseases in the human host. It is thus clear that microbes are important to human health, and many microbes present in the human organism can regulate host physiology and disease development [4,5].

A variety of organisms, such as *bacteria*, *fungi*, and *plants*, produce secondary metabolites, also known as natural products. Natural products have been a prolific source and an inspiration for numerous medical agents with widely divergent chemical structures and biological activities, including antimicrobial, immunosuppressive, anticancer, and anti-inflammatory activities, many of which have been developed as treatments and have potential therapeutic applications for human diseases [6]. In recent years, as the variety of drugs investigated by the medical field increases, the resistance of microbes has become more and more intense [7]. Previous research in the pharmaceutical industry has involved culturing some microbe species under greenhouse conditions and subsequently using them in drugs [8]. However, traditional wet lab experiments are costly and time-consuming, necessitating the urgent adoption of novel computational approaches to uncover potential relationships between microbes and drugs, thereby contributing to drug development analysis and human disease diagnosis.

In recent years, owing to the rapid advancement of bioinformatics, numerous distinguished researchers have constructed a series of databases concerning the associations between microbes and diseases, as well as microbes and drugs, greatly facilitating the computational analysis of potential relationships between microbes and drugs. For instance, Sun et al. established MDAD [9], which is a database consisting of 5505 associations between 180 microbes and 1388 drugs. Rajput et al. [10] developed the aBiofilm database, which records microbial resistance to drugs and includes biological, chemical, and structural details of 5027 antimicrobial agents. Andersen et al. [11] curated a dataset named DrugVirus, which includes 1281 associations between 118 compounds and 83 human viruses.

Based on these datasets, the application and development of various learning methods in bioinformatics have rapidly progressed, leading to the emergence of several computational models aimed at inferring potential microbe–drug associations. For instance, Anahtar et al. [12] explored the application of machine learning to the problem of antimicrobial resistance. However, the lack of high-quality training datasets resulted in suboptimal machine learning performance. Zhu et al. [13] proposed the HMDAKATZ method based on the KATZ measure. The method constructed a heterogeneous microbe–drug network and subsequently employed the Katz measure to calculate the correlation of nodes in this heterogeneous network. However, this method uses a simple measure, which fails to fully reflect similarity representation, thereby affecting the accuracy of MDA prediction. To include more node and edge information, Long et al. [14,15] introduced computational methods named GCNMDA and EGATMDA. GCNMDA is based on graph convolutional networks and conditional random fields with an attention mechanism to detect potential microbe-drug associations. The model primarily focuses on the first-order neighbors of nodes, neglecting the importance of higher-order neighbors, which limits the model’s performance. Deng et al. [16] designed a method called Graph2MDA, which predicts potential microbe–drug associations by constructing multimodal attribute graphs as input to a variational graph autoencoder to learn information from each node and the entire graph. Ma et al. [17] proposed a computational method named GACNNMDA for predicting associations between microbes and drugs. This method constructs two feature matrices and two heterogeneous microbe–drug networks, combining graph attention networks (GAT) and a convolutional neural network (CNN)-based classifier to predict potential microbe–drug associations. However, both of these methods have limitations in integrating multimodal attribute features.

Although previous methods have made progress in predicting microbe–drug associations, they still have limitations, such as neglecting higher-order neighbor information, inadequate handling of outlier nodes, and insufficient integration of multi-modal features. To improve prediction accuracy and model performance, this study proposes a model combining deep learning and machine learning: GCGACNN. This model integrates deep learning techniques, including Graph Convolutional Networks (GCN), Graph Attention Networks (GAT), and two-dimensional Convolutional Neural Networks (CNN), with the Random Forest algorithm. It comprehensively considers various aspects of microbes, drugs, and diseases to enhance the prediction of microbe–drug resistance associations. Specifically, we construct association and similarity matrices, extract features using deep graph convolutional networks, embed these features through graph attention mechanisms, integrate low-dimensional features using two-dimensional convolutional neural networks, and finally apply the Random Forest algorithm for prediction. This approach effectively combines the feature extraction capabilities of deep learning with the classification power of machine learning, significantly improving prediction performance. 

## 2. Materials and Methods

### 2.1. Data Sources

MDAD is a comprehensive database that integrates associations between microorganisms and drugs, encompassing 5505 validated records of interactions between 180 microorganisms and 1388 drugs. The microorganisms in this database include a variety of entities, such as *bacteria* and *fungi*, with primary information concentrated at the species level while also providing detailed information on specific strains. The drug-related information includes various chemical compounds and their effects on microorganisms, covering both direct antimicrobial agents and their targets. The research data in MDAD cover both acute and chronic bacterial infections, including classic antibiotic treatments as well as non-antibiotic drug applications.

In addition, we retrieved known associations among microorganisms, drugs, and diseases from the dataset compiled by Wang et al. [18]. This dataset includes 70,315 drug–disease associations and 15,633 microbe–disease associations. By filtering the disease data related to drugs and microorganisms in MDAD, we ultimately obtained 1121 unique drug–disease associations involving 233 drugs and 109 diseases and 402 distinct microbe–disease associations involving 73 microorganisms and 109 diseases.

Finally, we collected 2470 validated microbe–drug association records from a dataset compiled by Ma et al. [17], which includes 1373 drugs and 173 microorganisms. In constructing our dataset, we focused on the species level of microorganisms. Additionally, the drugs we studied include not only single chemical substances but also their potential mechanisms of action or drug combinations. The data encompass both acute and chronic forms of bacterial infections, thus comprehensively considering the complex interactions between microorganisms, drugs, and diseases. Our model still provides valuable predictions for microbe–drug associations at the species level. Future work will incorporate more detailed genetic information to further enhance the model’s predictive capabilities. Detailed information about these data are provided in Table 1. 

### 2.2. Overview

**Figure 1:**
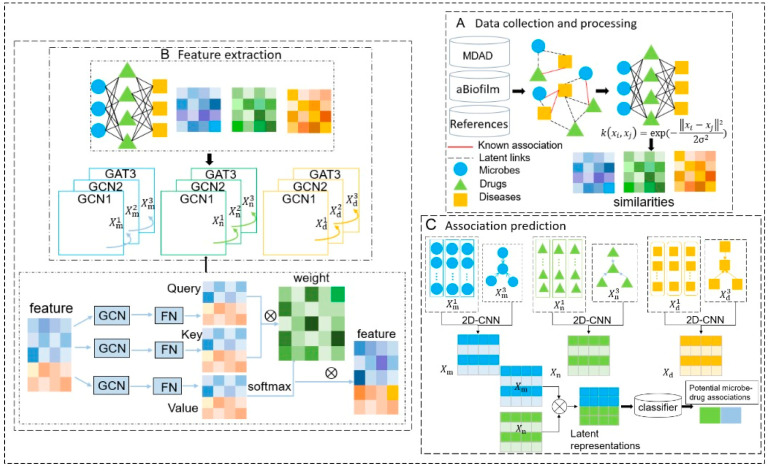
Flowchart of the GCGACNN. Part A shows data retrieval from MDAD and aBiofilm databases, constructing graph structures based on known microbe, drug, and disease associations, and evaluating node similarity. Part B demonstrates using GCN and GAT to extract multi-level node features and generate representations. Part C depicts integrating features via a 2D-CNN and predicting associations with a random forest algorithm.

As illustrated in Figure 1, the GCGACNN model primarily comprises three components:

Part A involves constructing the microbe–drug, microbe–disease, and drug–disease correlation matrices based on downloaded data associated with microbes, drugs, and diseases. Subsequently, three feature matrices are built utilizing these three correlation matrices.

Part B involves utilizing the feature matrices obtained from the initial step as inputs to the network layers. Graph convolutional layers and graph attention layers are employed to extract feature representations from various modalities. Ultimately, a two-dimensional convolutional neural network is used to fuse features and acquire effective representations.

Part C involves the introduction of a random forest-based classifier that employs learned embeddings to predict scores for microbe–drug associations.

### 2.3. Construct Association Matrices

Given a downloaded dataset encompassing m microbes, n drugs, d diseases, and their interconnections, our objective is to predict novel microbe–drug resistance associations by leveraging known associations among microbes, drugs, diseases, and their respective similarity characteristics. Firstly, based on the known microbe–drug interaction relationships, we construct a microbe–drug association matrix $A^{1}\in R^{m\times n}$. The construction rule is as follows: for any given microbe $m_{i}$ and drug $n_{j}$, if a known interaction relationship exists between them, then $A^{1}\left(i,j\right)=1$; otherwise, $A^{1}\left(i,j\right)=0$. Next, based on the known microbe–disease interaction relationships, we construct a microbe–disease association matrix $A^{2}\in R^{m\times d}$ using the same construction rule, where $A^{2}\left(i,j\right)=1$ if there is a known interaction, and $A^{2}\left(i,j\right)=0$ otherwise. Subsequently, based on the known drug–disease interaction relationships, we construct a drug–disease association matrix $A^{3}\in R^{n\times d}$ using a similar construction rule, where $A^{3}\left(i,j\right)=1$ if there is a known interaction, and $A^{3}\left(i,j\right)=0$ otherwise.

### 2.4. Similarity Calculation

We derive features related to microbes, drugs, and diseases based on the association matrices among these entities. Given the high sparsity of microbiome data, we employ the Gaussian Interaction Profile (GIP) [19] kernel function to calculate Gaussian kernel similarity between microbes and drugs in order to uncover more valuable similarity information. The GIP kernel function has been successfully applied for computing topological similarity between nodes, with the core idea that similar microbes (or drugs) interact in a similar manner to produce comparable interaction profiles. Specifically, in the microbe–drug association matrix, we posit that microbes with functional similarities exhibit analogous patterns of drug resistance. Two Gaussian similarity matrices, $G_{m}^{1}$ and $G_{m}^{2}$, are calculated for a given microbe employing the i-th row of the associated matrices $A^{1}$ and $A^{2}$. The computation of microbial similarity involves the utilization of a Gaussian kernel function, outlined as follows:

(1) $$G_{m}^{1}\left(i,j\right)=exp\left(-\alpha_{m}^{1}{\left\|A^{1}\left(i,:\right)-A^{1}\left(j,:\right)\right\|}^{2}\right)\;$$

(2) $$\alpha_{m}^{1}=\frac{1}{m}\sum_{k=1}^{m}{\left\|A^{1}\left(k,:\right)\right\|}^{2}$$

(3) $$G_{m}^{2}\left(i,j\right)=exp\left(-\alpha_{m}^{2}{\left\|A^{2}\left(i,:\right)-A^{2}\left(j,:\right)\right\|}^{2}\right)$$

(4) $$\alpha_{m}^{2}=\frac{1}{m}\sum_{k=1}^{m}{\left\|A^{2}\left(k,:\right)\right\|}^{2}$$

(5) $$G_{m}(i,j)=\frac{G_{m}^{1}\left(i,j\right)+G_{m}^{2}\left(i,j\right)}{2}$$

The given description entails that $A^{1}\in R^{m\times n}$ denotes the known microbe–drug resistance associations, while $A^{2}\in R^{m\times d}$ represents the known microbe–disease resistance associations. Additionally, $\left\|X\right\|$ signifies the Euclidean distance from *X* to the origin, with *m*, *n*, and *d* denoting the quantities of microbes, drugs, and diseases associated with the network, respectively. In this context, $G_{m}^{1}\left(i,j\right)$ stands for the similarity between two microbes based on their drug associations, with α serving as the kernel bandwidth parameter. The similarity $G_{m}^{2}\left(i,j\right)$ signifies the resemblance between two microbes based on their disease resistance associations. The interactive contour vectors, $A^{1}(k,:)$ and $A^{2}(k,:)$, are derived from the microbe associations of the i-th drugs and i-th diseases, respectively.

Similarly, the similarity between drugs is computed using the j-th column of matrix $A^{1}$ and the i-th row of matrix $A^{3}$.

(6) $$G_{n}^{1}\left(i,j\right)=exp(-\alpha_{n}^{1}{∥A^{1}(:,i)-A^{1}(:,j)∥}^{2})\;$$

(7) $$\alpha_{n}^{1}=\frac{1}{n}\sum_{k=1}^{n}{∥A^{1}(:,k)∥}^{2}$$

(8) $$G_{n}^{2}\left(i,j\right)=exp(-\alpha_{n}^{2}{∥A^{3}(i,:)-A^{3}(j,:)∥}^{2})\;$$

(9) $$\alpha_{n}^{2}=\frac{1}{n}\sum_{k=1}^{n}{∥A^{3}(k,:)∥}^{2}$$

(10) $$G_{n}(i,j)=\frac{G_{n}^{1}\left(i,j\right)+G_{n}^{2}\left(i,j\right)}{2}$$

$A^{3}\in R^{n\times d}$ signifies the established drug–disease resistance associations. The similarity assessment between two drugs, $n_{i}$ and $n_{j}$, based on their microbial associations denoted as $G_{n}^{1}$, is computed utilizing a Gaussian Interaction Profile (GIP) kernel with a bandwidth parameter, α. $G_{n}^{2}$ represents the similarity between two drugs based on their associations with diseases. To derive interaction profile vectors $A^{1}(:,k)$ and $A^{3}(k,:)$ effectively, they are based on the drug relevance of the j-th microbe and the drug relevance of the i-th disease, respectively.

Subsequently, we can compute the similarity of diseases using the j-th column of matrix $A^{2}$ and the j-th column of matrix $A^{3}$ as follows:

(11) $$G_{d}^{1}\left(i,j\right)=exp(-\alpha_{d}^{1}{∥A^{2}(:,i)-A^{2}(:,j)∥}^{2})\;$$

(12) $$\alpha_{d}^{1}=\frac{1}{d}\sum_{k=1}^{d}{∥A^{2}(:,k)∥}^{2}$$

(13) $$G_{d}^{2}\left(i,j\right)=exp(-\alpha_{d}^{2}{∥A^{3}(:,i)-A^{3}(:,j)∥}^{2})$$

(14) $$\alpha_{d}^{2}=\frac{1}{d}\sum_{k=1}^{d}{∥A^{3}(:,k)∥}^{2}$$

(15) $$G_{d}(i,j)=\frac{G_{d}^{1}\left(i,j\right)+G_{d}^{2}\left(i,j\right)}{2}$$

The GIP similarity between two diseases, $d_{i}$ and $d_{j}$, based on their microbe associations, was computed and denoted as $G_{d}^{1}\left(i,j\right)$. The similarity between two diseases based on their drug associations was computed and denoted as $G_{d}^{2}\left(i,j\right)$. The corresponding interactive contour vectors $A^{2}(:,k)$ and $A^{3}(:,k)$ were obtained based on the disease relevance of the j-th microbe and the disease relevance of the j-th drug.

Finally, we obtained similarity features $G_{m}\in R^{173\times 173}$ for microbes, $G_{n}\in R^{1373\times 1373}$ for drugs, and $G_{d}\in R^{109\times 109}$ for diseases. In calculating these similarity features, we addressed the high sparsity and compositional nature of microbiome data. Specifically, the Gaussian similarity matrix captures nonlinear relationships among microbes, drugs, and diseases, effectively extracting latent similarity information. The Gaussian kernel function measures distances between entities and maps them into a higher-dimensional space, overcoming challenges posed by data sparsity and revealing meaningful similarity patterns. Our approach takes into account the compositional nature of microbiome data, ensuring the effectiveness of the proposed Gaussian similarity matrices in this complex data context.

### 2.5. Embedding Learning

We designed a deep learning module that combines Graph Convolutional Networks (GCN) and Graph Attention Networks (GAT) to learn embeddings for microbes, drugs, and diseases. GCN performs convolution operations on the features of nodes and their neighbors to aggregate local information and generate context-rich node embeddings. GCN leverages normalized adjacency matrices to effectively propagate graph structural information and progressively extracts global features from local ones, providing comprehensive feature representations for subsequent tasks. Formally, given an undirected graph G with a node feature matrix X and an adjacency matrix A, the Graph Convolutional Network updates the node embeddings according to the following rule:

(16) $$H^{1}=ReLU({\overset{\sim }{A}}^{-\frac{1}{2}}\overset{\sim }{S}{\overset{\sim }{A}}^{-\frac{1}{2}}XW)$$

where $\tilde{S}=I+S$ and *I* represents the identity matrix, $\tilde{A}$ represents the degree matrix of matrix $\tilde{S}$, and W represents a trainable weight matrix. The processing through this layer yields the $H_{m}^{1},\;H_{n}^{1}$ and $H_{d}^{1}$. To better extract features for microbes, drugs, and diseases, we incorporate a Graph Attention Network layer into each module. GAT mitigates the issue of feature over-smoothing by applying attention weights to neighbor node features, and alleviates the problem of information over-squashing through its self-attention mechanism. Firstly, for any given node i in $H_{v}^{1}$ $\left(v=m,n,d\right)$, the computation of similarity coefficients with its adjacent nodes is as follows:

(17) $$e_{ij}=a(W\overset{\to }{H_{i}},W{\overset{\to }{H}}_{j})$$

where W is a trainable weight matrix, and a is a projection. Furthermore, the attention score $\alpha_{ij}$ between node i and node j would be calculated based on $e_{ij}$ according to the following formula:

(18) $$\alpha_{ij}=\frac{exp(e_{ij})}{\underset{t\neq i}{\sum }exp(e_{it})}$$

$\alpha_{ij}$ can be fully expanded as:

(19) $$\alpha_{ij}=\frac{exp\left(LeakReLU\left({\vec{a}}^{T}\left[W_{v}{\overset{\to }{H}}_{v_{i}}\left\|W_{v}{\overset{\to }{H}}_{v_{j}}\right\|W_{v}^{\prime }S_{v_{i}v_{j}}\right]\right)\right)}{\sum_{t\neq i}\left(LeakReLU\left({\vec{a}}^{T}\left[W_{v}{\vec{H}}_{v_{i}}\left\|W_{v}{\overset{\to }{H}}_{v_{t}}\right\|W_{v}^{\prime }S_{v_{i}v_{t}}\right]\right)\right)}$$

where $a^{T}$ is a weight vector and || is the concatenation operation. $W_{v}^{\prime }$ represents the weight of the edge $S_{ij}$. Based on this, we obtained the output feature as:

(20) $${\overset{\to }{H}}_{v}^{2^{\prime }}=ReLU\left(\sum_{j\neq i}\alpha_{ij}W_{v}{\overset{\to }{H}}_{i}\right)$$

where $H^{\prime }$ was further calculated by the multi-head attention mechanism as:

(21) $$\overset{\to }{H_{v}^{2}}={\left|\right|}_{k=1}^{k}ReLU\left(\sum_{j\neq i}\alpha_{ij}^{k}W^{k}{\overset{\to }{H}}_{i}\right)\;$$

The processing of this layer results in three distinct feature embeddings, namely $H_{m}^{2}$, $H_{n}^{2}$ and $H_{d}^{2}$. Next, we apply Graph Convolutional Networks (GCN) for more advanced feature extraction of node features. At this stage, the node features have been preliminarily processed by Graph Attention Networks (GAT), incorporating rich local interaction information. Through this stage, we aim to further enhance the aggregation effect of node features and integrate more comprehensive neighborhood information. The detailed implementation is as follows:

(22) $$H^{3}=ReLU({\overset{\sim }{A}}^{-\frac{1}{2}}\overset{\sim }{S}{\overset{\sim }{A}}^{-\frac{1}{2}}H^{2}W)$$

In order to achieve effective representation, the two-dimensional convolutional neural network was used to fuse the features of the above-mentioned feature matrix, and finally the effective representation was obtained.

### 2.6. Predicting Microbe–Drug Associations

After obtaining the feature embeddings for microbes *H_m_* and *H_n_* drugs, these embeddings can be used to generate the following matrices:

(23) $$A=H_{m}\times H_{n}^{T}$$

The predicted score matrix values represent the likelihood of the relationships. We trained the model using the binary cross-entropy (BCE) loss function, and the implementation details are as follows:

(24) Loss=−1n∑i=1n[yi⋅log(yi^)+(1−yi)⋅log(1−yi^)]

Random forests have demonstrated strong performance in binary relationship prediction. We utilize random forests as a classifier to predict the associations between microbiomes and drugs. For a new fused feature X, each decision tree $T_{i}$ provides a classification prediction result $H_{i}\left(X\right)$.

(25) $$H_{i}\left(X\right)={DecisionTree}_{i}\left(X\right)$$

The final classification result of the random forest is determined by the majority voting of all decision trees. Assuming there are K decision trees, the final prediction for category $\hat{y}$ is given by the following formula:

(26) $$\hat{y}={argmax}_{c}\sum_{i=1}^{k}1\left[H_{i}\left(X\right)=c\right]$$

where $1\left[\cdot \right]$ denotes the indicator function, which equals 1 if the condition is satisfied and 0 otherwise. The operator ${argmax}_{c}$ denotes the selection of the class c that maximizes the sum of the indicator functions. 

### 2.7. Model Training and Validation 

To demonstrate the practical application of GCGACNN in predicting microbe–drug relationships, we trained the model using real-word data. The training process involved several steps:

1. Data Preprocessing: We preprocessed the data by normalizing feature values and handling missing data through imputation techniques.
2. Feature Extraction: Using Gaussian kernel functions, we calculated similarity matrices for microbes, drugs, and diseases.
3. Model Training: We trained the GCGACNN model using the preprocessed data and extracted features. The model parameters were optimized using a grid search approach to find the best hyperparameters.
4. Validation: The model was validated using a five-fold cross-validation approach, and performance metrics such as AUC, AUPR, Accuracy, and F1-score were calculated to evaluate the model’s predictive performance.

## 3. Results

### 3.1. Comparison with State-of-the-Art Methods

In order to validate the prediction performance of GCGACNN, we compared GCGACNN with five existing microbe–drug association prediction methods, such as HMDAKATZ, GCNMDA, EGATMDA, Graph2MDA, and GACNNMDA. During the experimental process, we employed a control methodology similar to GACNNMDA, wherein the original parameters of all methods were fixed and executed on MDAD. These methods were evaluated using a five-fold cross-validation framework. Specifically, 20% of known associations and 20% of unvalidated potential associations were randomly selected as the test set, while the remaining 80% of known and unvalidated potential associations constituted the training set. Performance assessment was based on metrics including AUC, AUPR, Accuracy, and F1-Score.

(27) $$TPR=\frac{TP}{TP+FN}\;$$

(28) $$FPR=\frac{FP}{TN+FP}$$

(29) $$Precision=\frac{TP}{TN+FP}$$

(30) $$Recall=\frac{TP}{TP+FN}$$

(31) $$Accuracy=\frac{TP+TN}{TP+TN+FP+FN}$$

(32) $$F1-score=2\times \frac{Precision\times Recall}{Precision+Recall}$$

TP represents the number of samples correctly predicted as positive by the model, TN represents the number of samples correctly predicted as negative by the model, FP represents the number of negative class samples mistakenly predicted as positive by the model, and FN represents the number of positive class samples mistakenly predicted to be negative by the model. TPR, known as the True Positive Rate, denotes the proportion of positive class samples correctly predicted as positive by the model. FPR represents the proportion of negative class samples mistakenly predicted as positive. Precision signifies the proportion of samples predicted to be positive by the model that are actually positive. Accuracy indicates the proportion of samples correctly classified among the total samples. The F1-score is an indicator that comprehensively considers Precision and Recall. 

According to Table 2, the GCGACNN model exhibits outstanding performance, with an AUC value of 0.9853 ± 0.0026, surpassing the second-highest AUC value of 0.9777 ± 0.0109 from GACNNMDA by 0.76%. The AUPR value is 0.9860 ± 0.0028, showing a 4.8% improvement over the second-highest AUPR value of 0.9380 ± 0.0098 from Graph2MDA. The F1 score is 0.9385, significantly outperforming the F1 values of other models. However, in terms of accuracy, GCGACNN does not surpass these models. Nevertheless, GCGACNN can be considered a potential tool for predicting microbial-drug resistance associations.

### 3.2. Hyperparameter Sensitivity Analysis

**Figure 2:**
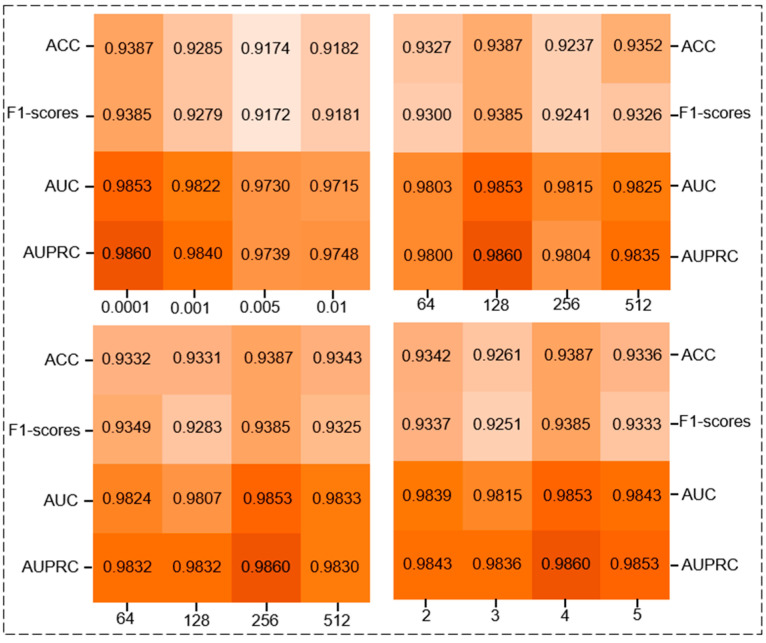
Influence of Different Hyperparameters on Model.

In this section, we investigated the sensitivity of parameters in GCGACNN, including the learning rate (LR) used during model training, the number of layers in GCN, the number of attention heads in GAT, the output channel sizes in the convolutional layers, and the feature dimensions for embedding. The overall results are illustrated in Figure 2. During the debugging process, we tested the learning rate (lr) within the range of {0.0001, 0.001, 0.005, 0.01}. As depicted in the figure, the model achieved optimal performance when lr was set to 0.0001, as shown in Table 3. For the convolutional layer’s output channel sizes, we conducted experiments within the range of {64, 128, 256, 512}. The graph illustrated that the model exhibited optimal performance when the channel size was set to 128, per the data depicted in Table 4. Exploring the impact of feature embedding dimensions, we varied the dimensions within the range of {64, 128, 256, 512}. The graph indicated that the model achieved its best performance when the feature embedding dimension was set to 256, according to the data presented in Table 5. Moreover, we examined the effect of the number of attention heads within the range of {2, 3, 4, 5}. As observed from the graph, the model performed optimally when the number of attention heads was set to 4, as shown in Table 6. 

### 3.3. Ablation Study

**Figure 3:**
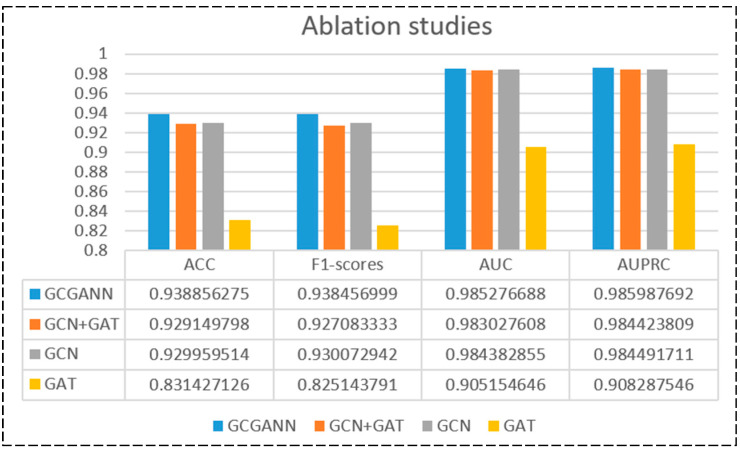
Results of the ablation experiments.

In this section, we will explore several variants of GCGANN to assess the significance of different components within the model. The detailed results are shown in Figure 3.

GCGANN with Single-layer GCN and GAT: We employ a single layer of GCN along with GAT for feature extraction, followed by concatenating these two features and passing them into convolutional layers for processing.

GCGANN without GAT: In this experiment, we omitted the GAT and utilized two layers of GCN for feature extraction. Subsequently, the two extracted features were concatenated and fed into convolutional layers for processing.

GCGANN without GCN: We utilize GAT for feature extraction without concatenation, directly passing it into convolutional layers for processing.

The results above indicate that using GCN alone outperforms the combination of GCN and GAT, while GCGACNN achieves the best results. This suggests that a reasonable combination of GCN and GAT can achieve better performance. Specifically, GCN effectively aggregates local information from neighboring nodes and captures global structural features between nodes. In contrast, GAT assigns different weights to neighboring nodes through a self-attention mechanism, thereby more accurately capturing complex interactions between nodes.

## 4. Discussion

This study further investigated the profound potential impact of microbial-drug interactions on human health. By combining deep learning and machine learning techniques, the GCGACNN model significantly outperformed some existing methods in terms of predictive performance. These findings are consistent with previous studies, highlighting the importance of advanced computational methods for understanding complex biological interactions. However, it is worth noting that although we used published databases of microbial, drug, and disease associations, these databases may not fully reflect the biological context. Therefore, the predictions made by the proposed deep learning method should be interpreted with caution. Future research should aim to further validate and expand these datasets to ensure the reliability and validity of the predictions.

Moreover, while the GCGACNN model demonstrates impressive predictive capabilities, there are still certain limitations in practical applications. Future work should focus on developing specific application guidelines and tools to help users effectively utilize this method in real-world scenarios. Through these efforts, we can better advance the field and provide stronger support for personalized medicine.

To date, numerous studies have provided substantial evidence for the profound potential impact of microbial-drug interactions on human health. Traditional culture-based methodologies indeed exhibit inherent limitations, particularly when confronted with intricate microbial assemblages. The advent of diverse computational methodologies has furnished us with a more comprehensive avenue for apprehending these interactions. Although numerous challenges persist at present, the momentum of development within this domain is swiftly accelerating. By amalgamating experimentally validated microbial-drug associations with advanced computational techniques, we envisage expediting the drug development process while simultaneously affording improved support for personalized medicine.

## 5. Conclusions

In this study, we proposed a computational model named GCGACNN for predicting potential associations between microbes and drugs. The GCGACNN model utilizes graph convolutional networks to learn latent representations of microbes and drugs and then obtains attention representations through graph attention networks. These integrated representations are processed through a two-dimensional convolutional neural network, and the final predictions are made using a random forest classifier. The main contributions of this study include:

1. Introducing known microbe–disease–drug interaction relationships in experiments and calculating their Gaussian similarity matrices.
2. Employing a stacked structure composed of multiple network layers to extract effective representations from the input similarity matrices.
3. Demonstrating the superior performance of the GCGACNN model compared to existing advanced methods in predicting potential microbe–drug associations.

Despite achieving satisfactory predictive performance, the model has limitations due to the sparse data structure. Future research should consider incorporating additional biological data to enrich the input features and construct multidimensional network data, aiming to enhance the predictive performance and generalizability of computational models.

## Figures and Tables

**Table 1:** Details of our downloaded data.

Type	Microbe	Drug	Disease
Microbe–drug associations	173	1373	-
Microbe–disease associations	173	-	109
Drug–disease associations	-	1373	109

**Table 2:** Results of various models. Bold indicates the optimal results.

Methods	Acc	F1-Scores	AUC	AUPR
HMDAKATZ	0.9774	0.3546	0.8712 ± 0.0010	0.2327 ± 0.0068
GCNMDA	0.9905	0.6672	0.9427 ± 0.0002	0.9133 ± 0.0031
EGATMDA	0.9081	0.6871	0.9585 ± 0.0053	0.9268 ± 0.0142
Graph2MDA	0.9934	0.7091	0.9567 ± 0.0039	0.9380 ± 0.0098
GACNNMDA	**0.9945**	0.7091	0.9777 ± 0.0109	0.7015 ± 0.0366
GCGACNN	0.9389	**0.9385**	**0.9853 ± 0.0026**	**0.9860 ± 0.0028**

**Table 3:** Metrics obtained with different learning rates. Bold indicates the optimal results.

LR	Acc	F1-Scores	AUC	AUPR
0.0001	**0.938856**	**0.938457**	**0.985277**	**0.985988**
0.001	0.928543	0.927933	0.982168	0.983911
0.005	0.917409	0.917243	0.972964	0.973854
0.01	0.918219	0.918069	0.971471	0.974806

**Table 4:** Metrics obtained with different output channel numbers. Bold indicates the optimal results.

Out Channels	Acc	F1-Scores	AUC	AUPR
64	0.932692	0.930027	0.980321	0.979949
128	**0.938856**	**0.938457**	**0.985277**	**0.985988**
256	0.923684	0.924114	0.981496	0.980357
512	0.935223	0.932632	0.982543	0.983517

**Table 5:** Metrics derived from different feature embedding dimensions. Bold indicates the optimal results.

Feature Embeddings	Acc	F1-Scores	AUC	AUPR
64	0.933198	0.934911	0.982393	0.983206
128	0.933126	0.928287	0.980677	0.98318
256	**0.938856**	**0.938457**	**0.985277**	**0.985988**
512	0.934247	0.932492	0.983271	0.983043

**Table 6:** Metrics derived from different attention mechanism heads. Bold indicates the optimal results.

LR	Acc	F1-Scores	AUC	AUPR
2	0.934211	0.933678	0.983868	0.984257
3	0.926113	0.925128	0.981495	0.98355
4	**0.938856**	**0.938457**	**0.985277**	**0.985988**
5	0.933603	0.933335	0.984288	0.985313

## Data Availability

The datasets analyzed during the current study are available from the corresponding author on reasonable request.

## References

[1] Wen Z., Yan C., Duan G., Li S., Wu F.X., Wang J. (2021). A survey on predicting microbe-disease associations: Biological data and computational methods. Brief. Bioinform..

[2] Clemente J.C., Ursell L.K., Parfrey L.W., Knight R. (2012). The impact of the gut microbiota on human health: An integrative view. Cell.

[3] Kumar A., Chordia N. (2017). Role of microbes in human health. Appl. Microbiol. Open Access.

[4] Dethlefsen L., McFall-Ngai M., Relman D.A. (2007). An ecological and evolutionary perspective on human–microbe mutualism and disease. Nature.

[5] Petrosino J.F. (2018). The microbiome in precision medicine: The way forward. Genome Med..

[6] Pham J.V., Yilma M.A., Feliz A., Majid M.T., Maffetone N., Walker J.R., Kim E., Cho H.J., Reynolds J.M., Song M.C. (2019). A review of the microbial production of bioactive natural products and biologics. Front. Microbiol..

[7] Ramirez M., Rajaram S., Steininger R.J., Osipchuk D., Roth M.A., Morinishi L.S., Evans L., Ji W., Hsu C.H., Thurley K. (2016). Diverse drug-resistance mechanisms can emerge from drug-tolerant cancer persister cells. Nat. Commun..

[8] Pammolli F., Magazzini L., Riccaboni M. (2011). The productivity crisis in pharmaceutical R&D. Nat. Rev. Drug Discov..

[9] Sun Y.Z., Zhang D.H., Cai S.B., Ming Z., Li J.Q., Chen X. (2018). MDAD: A special resource for microbe-drug associations. Front. Cell. Infect. Microbiol..

[10] Rajput A., Thakur A., Sharma S., Kumar M. (2018). aBiofilm: A resource of anti-biofilm agents and their potential implications in targeting antibiotic drug resistance. Nucleic Acids Res..

[11] Andersen P.I., Ianevski A., Lysvand H., Vitkauskiene A., Oksenych V., Bjørås M., Telling K., Lutsar I., Dumpis U., Irie Y. (2020). Discovery and development of safe-in-man broad-spectrum antiviral agents. Int. J. Infect. Dis..

[12] Anahtar M.N., Yang J.H., Kanjilal S. (2021). Applications of machine learning to the problem of antimicrobial resistance: An emerging model for translational research. J. Clin. Microbiol..

[13] Zhu L., Duan G., Yan C., Wang J. (2019). Prediction of microbe-drug associations based on Katz measure. Proceedings of the 2019 IEEE International Conference on Bioinformatics and Biomedicine (BIBM).

[14] Long Y., Wu M., Kwoh C.K., Luo J., Li X. (2020). Predicting human microbe–drug associations via graph convolutional network with conditional random field. Bioinformatics.

[15] Long Y., Wu M., Liu Y., Kwoh C.K., Luo J., Li X. (2020). Ensembling graph attention networks for human microbe–drug association prediction. Bioinformatics.

[16] Deng L., Huang Y., Liu X., Liu H. (2022). Graph2MDA: A multi-modal variational graph embedding model for predicting microbe–drug associations. Bioinformatics.

[17] Ma Q., Tan Y., Wang L. (2023). GACNNMDA: A computational model for predicting potential human microbe-drug associations based on graph attention network and CNN-based classifier. BMC Bioinform..

[18] Wang L., Tan Y., Yang X., Kuang L., Ping P. (2022). Review on predicting pairwise relationships between human microbes, drugs and diseases: From biological data to computational models. Brief. Bioinform..

[19] Luo J., Long Y. (2018). NTSHMDA: Prediction of human microbe-disease association based on random walk by integrating network topological similarity. IEEE/ACM Trans. Comput. Biol. Bioinform..

